# Impact of cardiovascular magnetic resonance on management and clinical decision-making in heart failure patients

**DOI:** 10.1186/1532-429X-15-89

**Published:** 2013-10-01

**Authors:** Siddique A Abbasi, Andrew Ertel, Ravi V Shah, Vineet Dandekar, Jaehoon Chung, Geetha Bhat, Ankit A Desai, Raymond Y Kwong, Afshin Farzaneh-Far

**Affiliations:** 1 Division of Cardiovascular Medicine, Department of Medicine, Brigham and Women’s Hospital and Harvard Medical School, Boston, MA, USA; 2 Section of Cardiology, Department of Medicine, University of Illinois at Chicago, 840 South Wood St. M/C 715, Suite 920 S, Chicago, IL 60612, USA; 3 Division of Cardiology, Department of Medicine, Massachusetts General Hospital and Harvard Medical School, Boston, MA, USA; 4 Center for Heart Transplant and Assist Devices, Advocate Christ Medical Center, Oak Lawn, IL, USA; 5 Department of Radiology, University of Illinois at Chicago, Chicago, IL, USA

## Abstract

**Background:**

Cardiovascular magnetic resonance (CMR) can provide important diagnostic and prognostic information in patients with heart failure. However, in the current health care environment, use of a new imaging modality like CMR requires evidence for direct additive impact on clinical management. We sought to evaluate the impact of CMR on clinical management and diagnosis in patients with heart failure.

**Methods:**

We prospectively studied 150 consecutive patients with heart failure and an ejection fraction ≤50% referred for CMR. Definitions for “significant clinical impact” of CMR were pre-defined and collected directly from medical records and/or from patients. Categories of significant clinical impact included: new diagnosis, medication change, hospital admission/discharge, as well as performance or avoidance of invasive procedures (angiography, revascularization, device therapy or biopsy).

**Results:**

Overall, CMR had a significant clinical impact in 65% of patients. This included an entirely new diagnosis in 30% of cases and a change in management in 52%. CMR results directly led to angiography in 9% and to the performance of percutaneous coronary intervention in 7%. In a multivariable model that included clinical and imaging parameters, presence of late gadolinium enhancement (LGE) was the only independent predictor of “significant clinical impact” (OR 6.72, 95% CI 2.56-17.60, p=0.0001).

**Conclusions:**

CMR made a significant additive clinical impact on management, decision-making and diagnosis in 65% of heart failure patients. This additive impact was seen despite universal use of prior echocardiography in this patient group. The presence of LGE was the best independent predictor of significant clinical impact following CMR.

## Background

Cardiovascular magnetic resonance (CMR) is emerging as a powerful diagnostic tool capable of assessing a wide array of important variables in heart failure [1,2], including cardiac structure, function, viability, scar pattern, perfusion, and the presence of thrombus [3-10].The versatility of CMR in assessing the etiology and prognosis of cardiomyopathies makes it a potentially valuable adjunct to traditional imaging modalities [11].

Current guidelines from the Heart Failure Society of America recommend using CMR in the diagnosis of amyloidosis, sarcoidosis, hemochromatosis, and possibly in viability assessment. However, there is no particular recommendation for its use in initial evaluation of heart failure [12]. In the current health care environment, use of a new imaging modality like CMR requires evidence for direct additive impact on clinical management. We hypothesized that findings on CMR would significantly alter management, clinical decision-making and diagnoses in patients referred from an advanced heart failure program. Furthermore, we sought to assess which findings on CMR best predict subsequent clinical impact.

## Methods

### Study protocol

This was a prospective observational study from an academic medical center in the United States with an established heart failure program. We studied 150 consecutive patients referred for CMR from the heart failure program over a period of 6-months. Patients were included if they had an LVEF≤50% by prior imaging studies. CMR was performed by standardized protocols recommended by the Society of Cardiovascular Magnetic Resonance (SCMR) [13] on 1.5T or 3T scanners and interpreted by an experienced reader (AF). The CMR protocol included ventricular function assessment (by a standard steady-state free precession sequence obtained in short and long-axis views) and late gadolinium enhancement (LGE) imaging obtained 10-15 minutes after administration of intravenous gadolinium (0.15 mmol/kg), as per published guidelines [13]. The presence and location of LGE was determined by visual inspection using the AHA 17-segment model [14]. Regional enhancement was scored according to the spatial extent of enhanced tissue within each segment (0 = no enhancement; 1 = 1%–25% enhanced; 2 = 26%–50%; 3 = 51%–75%; and 4 = 76%–100%) [14]. The pattern of enhancement was classified as either Coronary Artery Disease (CAD)-type or non-CAD-type on the basis of subendocardial involvement [15,16]. Demographic and clinical characteristics were recorded at the time of CMR, including age, gender, New York Heart Association (NYHA) functional class, left ventricular ejection fraction (LVEF), medications, previous cardiac imaging, and referral indications. The study was approved by the local institutional review board, and all subjects gave written informed consent.

### Influence of CMR on subsequent clinical management

**Figure 1:**
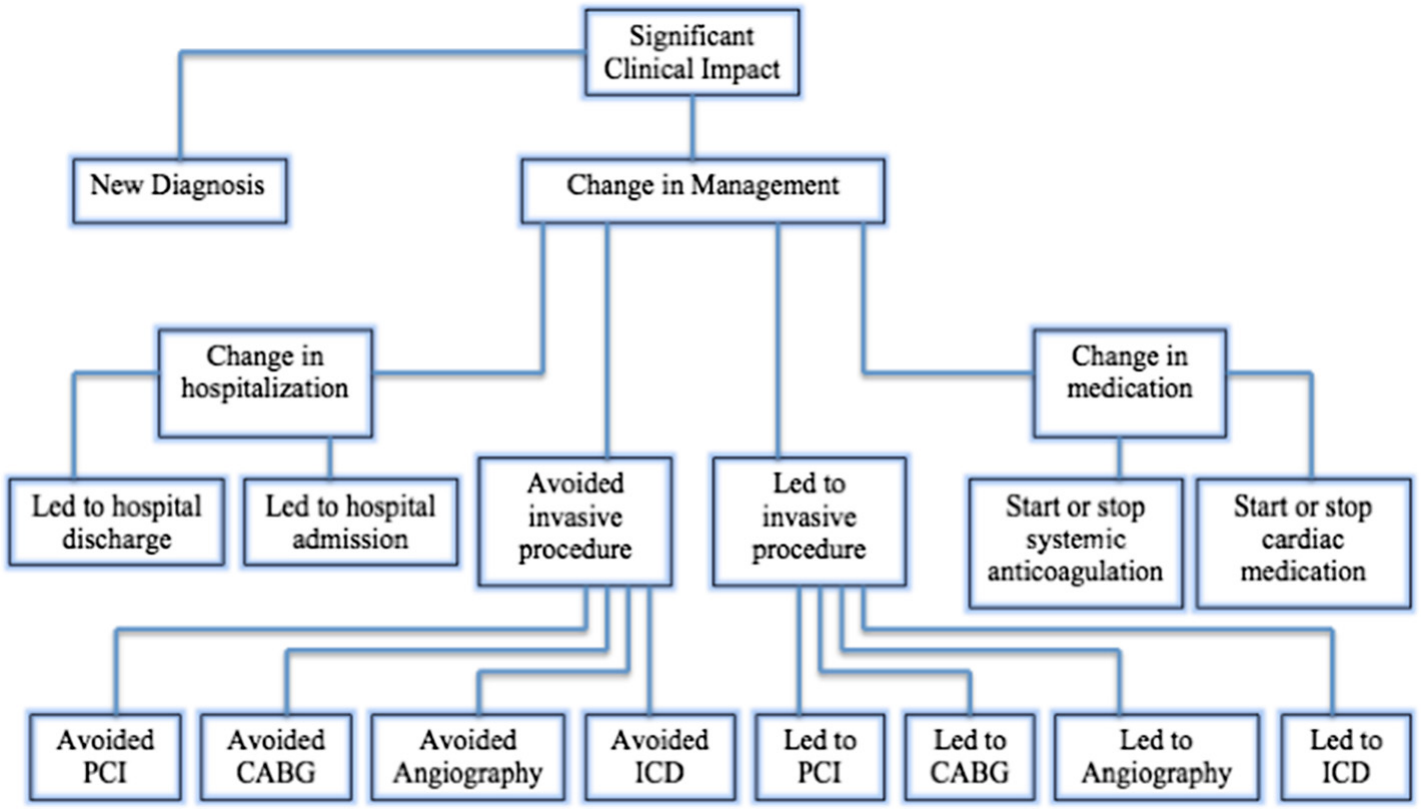
**Definition of Significant Clinical Impact.** PCI=percutaneous coronary intervention; CABG=coronary artery bypass grafting; ICD=implantable cardioverter-defibrillator.

Definitions for “significant clinical impact” of CMR were pre-defined and were collected directly from medical records or patient interview (Figure 1). Data was collected and interpreted by one of the primary investigators in all cases. Another physician, unaffiliated with the CMR or heart failure programs, independently interpreted a randomly generated sample of the patients. This physician was blinded to the previous interpretations of “significant clinical impact.” A third independent physician adjudicated any discrepancy between the interpreters.

#### ***Significant clinical impact***

“Significant Clinical Impact” was defined as an entirely “new diagnosis” or if a “change in management” occurred (see definitions below and Figure 1). A patient with both a new diagnosis and a change in management (e.g. CMR findings led to the new diagnosis of hypertrophic cardiomyopathy and the patient received an ICD) was only counted once toward “significant clinical impact.”

#### ***New diagnosis***

**Figure 2:**
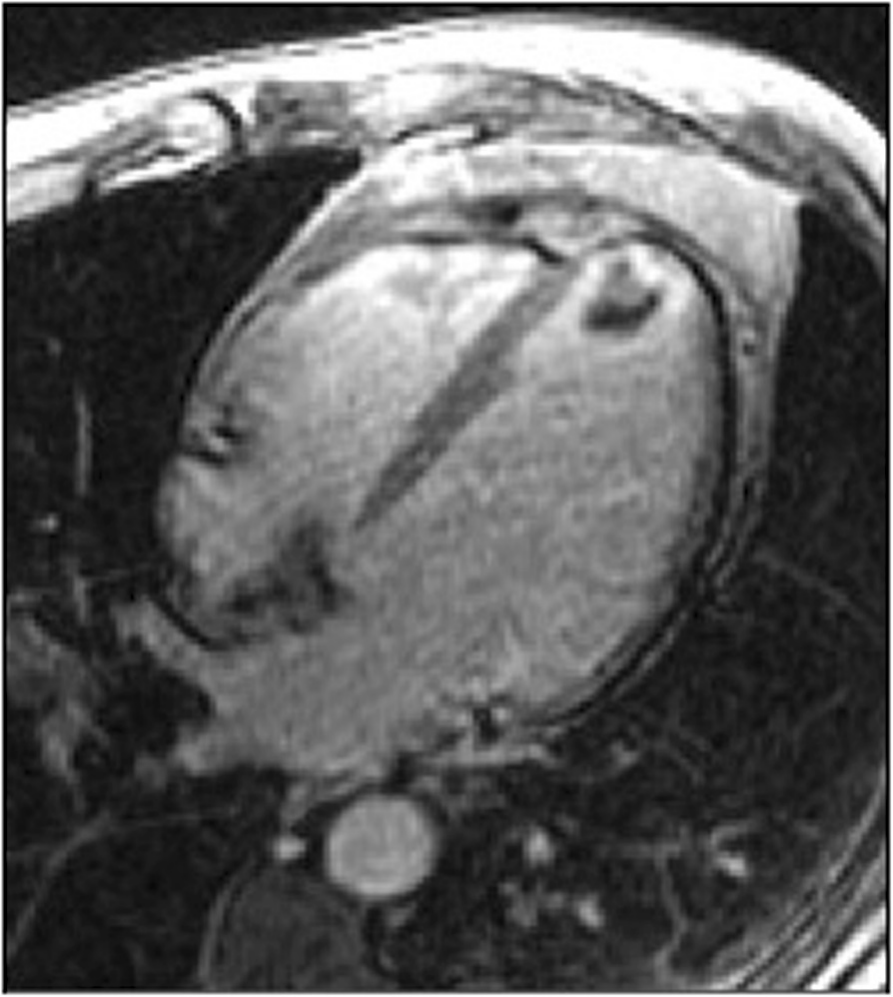
**Example of a New Diagnosis.** A 32 year-old woman with sickle cell anemia was referred for evaluation of iron overload by T2* imaging, which was normal. However, nearly transmural hyperenhancement (white arrows) was seen in the apical inferior wall on late enhancement imaging, indicative of previously unrecognized myocardial infarction.

A “new diagnosis” was defined as occurring only if it was previously unknown by the referring physician. For example, a patient who had a CMR for assessment of iron overload but was found instead to have evidence of myocardial infarction would be defined as having had a new diagnosis (Figure 2).

#### ***Change in management***

**Figure 3:**
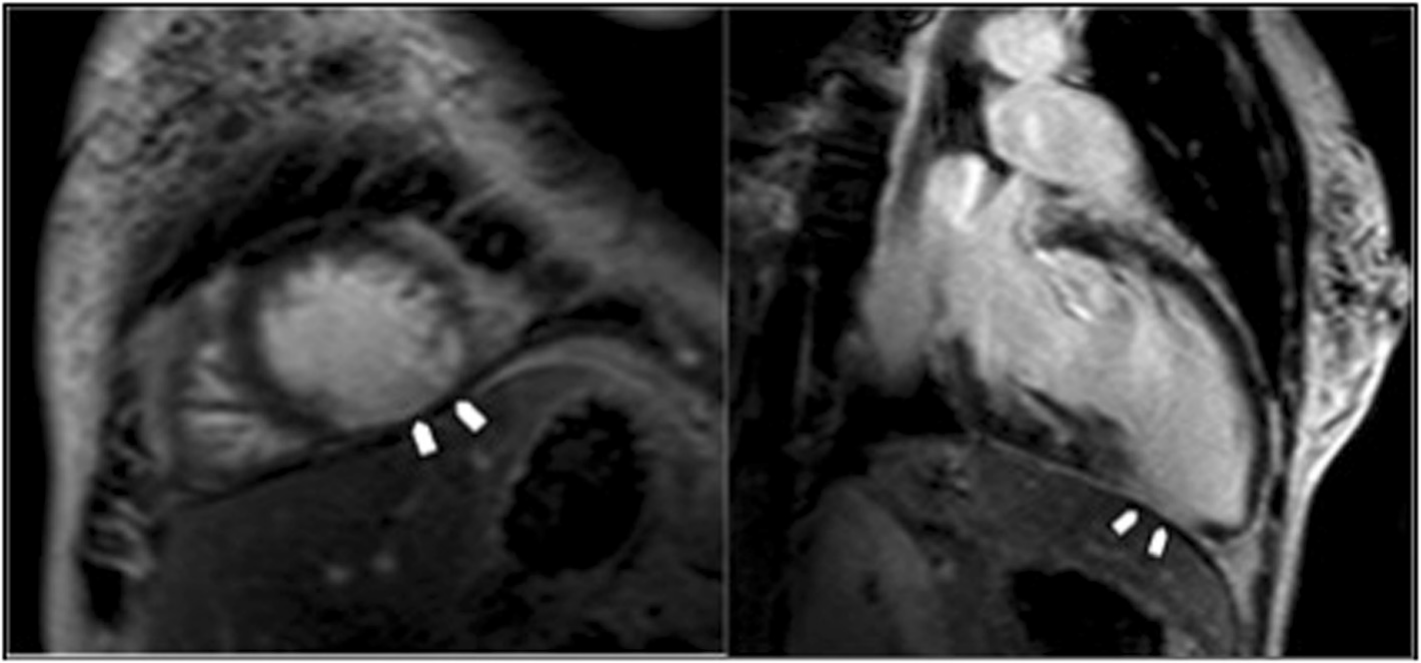
**Example of a Change in Management.** A 65 year-old man was referred for assessment of ventricular function and viability testing. CMR unexpectedly revealed a large apical thrombus, for which the patient was admitted to hospital for initiation of systemic anticoagulation.

“Change in Management” was defined as occurring if one of the following criteria were met: CMR results led directly to the performance of or avoidance of an invasive procedure (endomyocardial biopsy, angiography, PCI, CABG, or ICD placement); CMR findings led to the initiation or discontinuation of a cardiac medication; CMR results led to the initiation or cessation of systemic anticoagulation; or CMR findings directly resulted in hospital admission or discharge (Figure 1). A patient whose management was changed in more than one category (e.g. CMR findings led to the new diagnosis of intracardiac thrombus and the patient was subsequently admitted to the hospital for intravenous anticoagulation) was only counted once toward “significant clinical impact” (Figure 3).

### Statistical analysis

Normally distributed data are presented as the mean ± SD. Continuous covariates were compared by Student’s t-test or Wilcoxon rank-sum (depending on data normality). Fishers exact test was used to compare discrete data between groups. To identify which clinical and CMR indices were associated with a “significant clinical impact”, we performed univariable (unadjusted) logistic regression analysisto estimate the unadjusted odds ratios (OR) and the 95% confidence intervals (CIs) for the following variables: age, gender, NYHA class,diabetes, hypertension, prior revascularization, LVEF and presence of LGE. For the multivariable model, candidate variables showing a possible association with “significant clinical impact” by univariable analysis (p < 0.10) were considered one-at-a-time starting with the most significant variable. Significant variables were determined by stepwise selection (and backward elimination) at the 0.05 level of significance. All analyses were performed in MedCalc (version 12.3, New York City, NY) or SAS (version 9.3, SAS Institute, Cary, NC).

## Results

### Baseline clinical characteristics

Mean age was 54 years, with 57% of patients being men (Table 1). The mean LVEF was 38% (±11%). A 3.0 Tesla magnet was used in 51% of the studies. All patients had a recent echocardiogram prior to their CMR study. Of the 20 patients randomly selected for secondary review by an interpreter unaffiliated with the CMR and heart failure programs, there was only one instance of discrepant interpretation. A third independent physician adjudicated the discrepancy in this single case. Image quality was qualitatively assessed as good vs poor at the time of reading. Overall there were 7 patients (5% of cohort) with poor image quality. Of these, 6 patients were performed at 3Tesla and 4 patients had atrial fibrillation. Overall 17% (26 patients) of the total cohort had atrial fibrillation and image quality was assessed as good in the majority of these (22 cases or 85%).

**Table 1:** Study Population

**Baseline characteristics**	
**Sex**	
**Male**	57%
**Female**	43%
**Age (years±SD)**	54 (±17)
**Family history of dilated cardiomyopathy**	10%
**Family history of CAD**	18%
**NYHA class**	
**I**	26%
**II**	49%
**III**	24%
**IV**	1%
**Ejection fraction (±SD)**	38% (±11%)
**History of alcohol excess**	7%
**Preceding flu-like illness**	8%
**Comorbidities**	
**Diabetes**	28%
**Hypertension**	75%
**Atrial fibrillation**	17%
**Muscular dystrophy**	3%
**Prior revascularizations**	
**CABG**	7%
**PCI**	29%
**Cardiac diagnostics**	
**Prior SPECT**	18%
**Prior echocardiogram**	100%
**Prior coronary angiogram**	59%
**Referral indications**	
**Assessment of cardiomyopathy of unknown etiology**	59%
**Assessment of viability**	31%
**Assessment of suspected myocarditis**	5%
**Other**	5%

*CAD*=Coronary artery disease; *NYHA*=New york heart association; *PCI*=Percutaneous coronary intervention; *CABG*=Coronary artery bypass grafting; *SPECT*=Single photon emission computed tomography.

### Indications for CMR

The most common referral indications were for assessment of: 1) cardiomyopathy of unknown etiology, 2) viability, 3) suspected myocarditis (Table 1).

### New diagnoses

**Figure 4:**
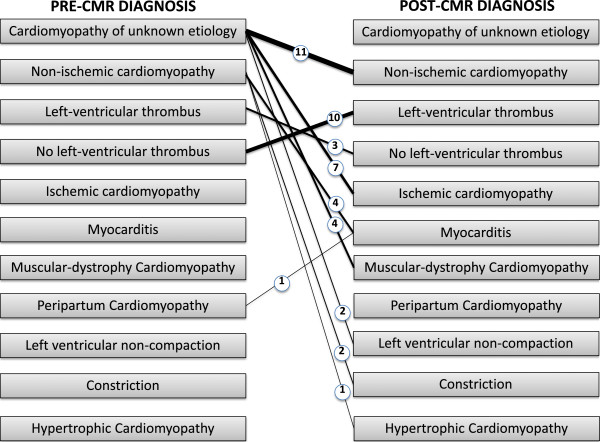
**Change in diagnosis after performance of CMR.** Weighted lines represent number of patients (also numerically represented within the circle).

An entirely new diagnosis was found in 30% of cases (Figure 4). The most frequently discovered new diagnoses were non-ischemic cardiomyopathy – based on scar pattern (found in 7% of all cases, accounting for 24% of new diagnoses), left-ventricular thrombus (found in 6.7% of all cases, accounting for 22% of new diagnoses), and coronary artery disease (found in 5% of all cases, accounting for 16% of new diagnoses). Rarer diagnoses included myocarditis and muscular dystrophy related cardiomyopathy. The patients that were diagnosed with myocarditis had a flu like prodrome with chest pain, elevated cardiac enzymes, normal or non-obstructive coronary arteries on angiography and a mid-myocardial or epicardial LGE pattern on CMR [17]. The patients that were diagnosed with muscular dystrophy related cardiomyopathy had a known neurological diagnosis of muscular dystrophy and were found to have midwall or epicardial LGE on CMR in the basal inferior and/or infero-lateral walls [18]. Overall, in more than half of cases (57%), the discovery of a new diagnosis led to a change in patient management.

### Change in management

The results of a patient’s CMR led to a change in management in a variety of ways (Table 2). The most commonly impacted elements of patient care were catheter-based procedures, where 27% of patients had their care affected directly as a result of CMR findings (Table 2). CMR results led to angiography in 9% of cases and led to the avoidance of angiography in 11% of cases. CMR findings led to PCI in 7% of patients and led to the avoidance of PCI in 5% of cases. CMR findings also had significant impact on the role of surgical revascularization, where the results of a patient’s CMR led to CABG in 5% of cases and led to the avoidance of CABG in 5% of cases. The impact of CMR on these procedures was principally based on LGE findings. The absence of viability led to avoidance of revascularization in that arterial territory. Depending on the arterial territories involved, this sometimes led to avoidance of revascularization altogether or to switching from revascularization with CABG to PCI. In patients being evaluated for cardiomyopathy of unknown etiology, the absence of LGE at a young age (all less than 30 years old) without CAD risk factors, and history or ECG evidence of MI, led to clinicians deciding to avoid coronary angiography [19].

**Table 2:** Impact of CMR on patient management

	
**Impact on catheter-based procedures**	41 (27%)
**Led to angiography**	14 (9%)
**Avoided angiography**	17 (11%)
**Led to biopsy**	3 (2%)
**Avoided biopsy**	1 (1%)
**Led to PCI**	11 (7%)
**Avoided PCI**	8 (5%)
**Impact on surgical revascularization**	14 (10%)
**Led to CABG**	7 (5%)
**Avoided CABG**	7 (5%)
**Impact on electrophysiology procedures**	15 (10%)
**Led to ICD**	5 (3%)
**Avoided ICD**	10 (7%)
**Impact on medications**	25 (17%)
**Started anticoagulation**	9 (6%)
**Stopped anticoagulation**	9 (6%)
**Started cardiac medication**	4 (3%)
**Stopped cardiac medication**	3 (2%)
**Hospital admission**	2 (1%)
**Hospital discharge**	2 (1%)

*PCI*=Percutaneous coronary intervention; *CABG*=Coronary artery bypass grafting; *ICD*=Implantable cardioverter-defibrillator.

Medication management was also significantly affected by the results of CMR testing (Table 2). Systemic anticoagulation was initiated in 6% of cases on account of thrombus imaging on CMR, and systemic anticoagulation was stopped in 6% of cases due to absence of thrombus on CMR despite being suspected by echocardiography (CMR was performed within 24 hours of the echo in all of these cases). Other cardiac medications were initiated in 3% of cases and discontinued in 2% based upon CMR findings.

### Significant clinical impact

**Figure 5:**
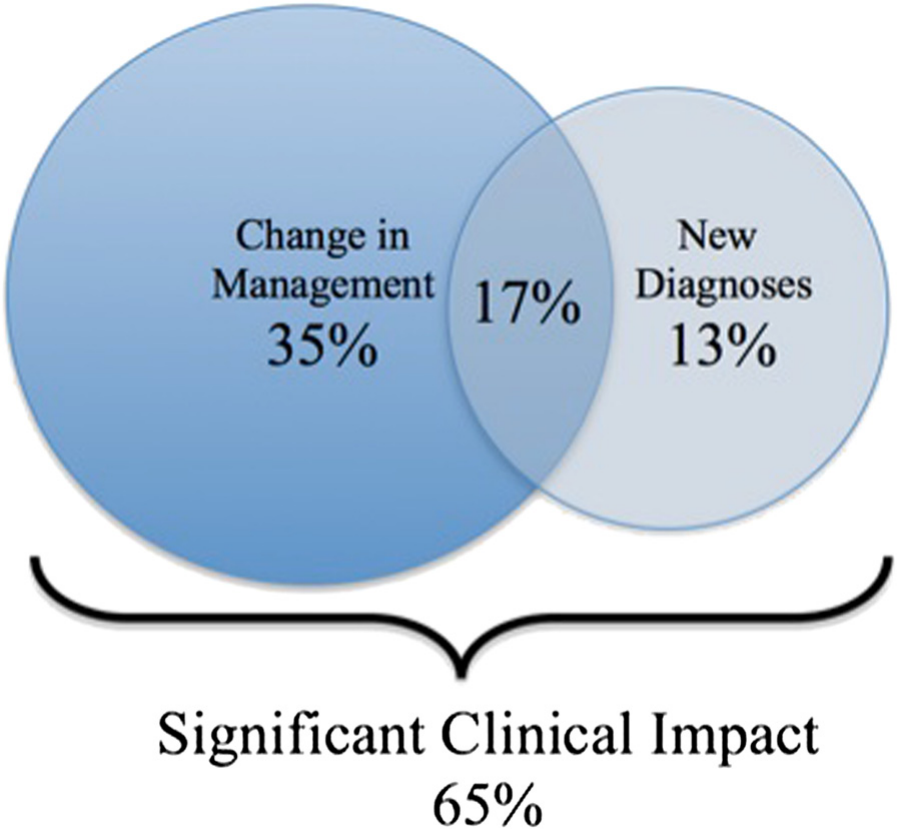
**Significant Clinical Impact of CMR.** On the basis of CMR findings, 52% of patients had a change in management and 30% of patients had a new diagnosis. In 17% of patients CMR resulted in both a change in management and a new diagnosis. In total, CMR had a significant clinical impact on 65% of patients.

There was a significant clinical impact in nearly two-thirds of cases (65%) overall, which included a new diagnosis in 30% of cases and a change in management in 52% of cases (Figure 5). A total of 17% of patients had both a new diagnosis and a change in management; however, in accordance to our pre-defined categories, these patients were counted only once toward our measurement of significant clinical impact.

Gender (OR 2.31, 95% CI 1.17-4.58, p=0.02), LVEF (OR 0.97, 95% CI 0.94-1.00, p=0.03) and LGE (OR 7.06, 95% CI 3.01-16.56, p<0.0001) were significant univariable predictors of significant clinical impact. In a multivariable model adjusting for clinical and imaging parameters, only LGE (OR 6.72, 95% CI 2.56-17.60, p=0.0001) independently predicted significant clinical impact (Table 3).

**Table 3:** Predictors of significant clinical impact

	**UNIVARIABLE**			**MULTIVARIABLE**		
Parameter	Odds ratio	95% CI	P-value	Odds ratio	95% CI	P-value
**Gender**	2.31	1.17-4.58	0.02	1.16	0.51-2.61	0.72
**Age**	1.02	1.02-1.04	0.10	1.01	0.99-1.04	0.32
**NYHA class**	1.20	0.76-1.88	0.44	0.73	0.41-1.28	0.27
**LVEF**	0.97	0.94-1.00	0.03	0.98	0.95-1.10	0.23
**Diabetes**	1.31	0.61-2.81	0.48	1.22	0.48-3.10	0.67
**Hypertension**	1.71	0.81-3.63	0.16	0.87	0.30-2.59	0.81
**Prior revascularization**	1.84	0.93-3.63	0.08	0.89	0.36-2.21	0.80
**LGE**	7.06	3.01-16.56	<0.0001	6.72	2.56-17.60	0.0001

Univariable and multivariable associations of clinical and imaging parameters with subsequent significant clinical impact. *NYHA* = New York Heart Association, *LVEF* = Left Ventricular Ejection Fraction, *LGE* = Late Gadolinium Enhancement, *CI* = Confidence Interval.

## Discussion

To our knowledge, this is the first study systematically investigating the clinical impact of CMR on management and diagnosis in heart failure patients in daily practice.

### Comparison with the EuroCMR Registry

The EuroCMR registry was a multi-center registry that followed 11,040 patients from 20 European centers and sought to evaluate indications, image quality, safety, and impact on patient management of routine CMR [20]. The investigators found that in 61.8% of patients, CMR had a significant impact on management, pre-defined as findings that had therapeutic consequence or led to an entirely new diagnosis. In addition, the EuroCMR registry reported discovery of a completely new diagnosis in 16.8% of cases. However, there are important differences between our study and the EuroCMR registry. Our study is the first to look specifically at heart failure patients. The EuroCMR registry performed subgroup analyses on patient age, indication, and the performance of stress testing; however, no specific analysis was performed on heart failure patients or those with depressed LVEF. Within our study, all patients had previously undergone testing with echocardiography, whereas in the EuroCMR registry, CMR was the first test performed in 23.1% of patients. Importantly, in our study we examined the clinical and CMR variables which were most predictive of significant clinical impact using multivariable modeling. Another difference between the two registries was that the EuroCMR study included patients undergoing stress testing (in 20.9% of cases). We did not include stress CMR in this study because it is currently significantly less available and we wanted the study results to be as widely applicable as possible. It is possible that inclusion of stress CMR would lead to greater impact on heart failure patient management. In our study we attempted to reduce bias in interpretation of what constitutes “significant clinical impact” by using additional independent physician interpreters who were unaffiliated with the CMR program. Finally, in contrast to the EuroCMR registry, our study sheds specific light on the effects of CMR within the context of the US healthcare system.

### Implications

Currently many physicians reserve the use of CMR in heart failure only for a small subset of 'boutique’ indications. Guidelines from the Heart Failure Society of America recommends the use of CMR in the diagnosis of amyloidosis, sarcoidosis, hemochromatosis, and possibly in viability assessment [12]. However, an increasing amount of data has emerged suggesting that CMR is an effective initial test in the evaluation of patients presenting with heart failure [1,2,19]. Our observations lend support to this notion.

Our study shows that CMR has significant clinical impact in this population despite universal use of prior echocardiography. Given its accessibility and low cost, we believe that echocardiography remains an essential first-line test in these patients. Our purpose was not to compare the diagnostic ability of echocardiography with CMR but rather to assess the additive clinical value of CMR in heart failure patients that underwent standard diagnostic evaluations (which invariably included echo). However, further studies are needed to support such an approach. In particular, there is a need for larger, multi-center studies that evaluate not only significant clinical impact, but also outcomes and cost-effectiveness.

### Limitations

Our study was limited by a small sample size (150 patients) drawn from a single academic tertiary center. As a result, there was undoubtedly significant selection bias present and the patient group may not be representative of the wider heart failure population. For example, the mean age of our study population was 54 years, which is significantly younger than that reported in the community [21]. Furthermore, it is probable that clinicians selected patients that were more likely to benefit from CMR. This is consistent with the observation that the total number of patients referred for CMR in this study represents only a small proportion (approximately 30%) of the overall population with heart failure and depressed LVEF passing through the heart failure program during the study period.

Another important limitation is that this study was not designed to test the validity of the management decisions or new diagnoses, which occurred as a result of the CMR. A change in management or a new diagnosis may not necessarily equate to improved outcomes. This study was not designed to test the evidence base on which clinicians made management decisions. For example, two recent studies have questioned the value of revascularization in patients with ischemic cardiomyopathy [22,23]. It is also possible that CMR driven changes in management and new diagnoses resulted in further testing and/or procedures downstream, some of which may be associated with their own inherent risks. To address these questions, future randomized prospective studies are needed to compare outcomes between a CMR guided vs conventional approach in heart failure patients.

In this study, patient treatment was entirely at the discretion of the patient’s cardiologist and, therefore, there may be significant management differences between individuals that impacted the results. Our study did not analyze cost-effectiveness of CMR as compared to current testing in the heart failure population. Cost-effectiveness analyses will be needed to better define the possible benefits of CMR in the management of heart failure. Although it maybe argued that interpretation of “significant clinical impact,” in our study was subjective, we attempted to minimize this by predefining all categories and using several independent interpreters.

Lastly, it is important to realize that there are limitations to the use of CMR in heart failure patients. These include the additional costs of the procedure, long scan times, as well as difficulties in scanning individuals with claustrophobia and severe orthopnea. In addition, a significant portion of heart failure patients have relative or absolute contraindications to CMR, including severe renal impairment and implanted devices such as pacemakers, defibrillators and ventricular assist devices.

## Conclusions

CMR makes a significant impact on clinical management, decision-making and diagnosis in 65% of selected heart failure patients. This additive impact was seen despite universal use of prior echocardiography in this patient group. Our study lends support to the growing use of CMR in the assessment of heart failure patients.

## Abbreviations

CABG: Coronary artery bypass grafting; CAD: Coronary artery disease; CMR: Cardiovascular magnetic resonance; HCM: Hypertrophic cardiomyopathy; ICD: Implantable cardioverter-defibrillator; LGE: Late gadolinium enhancement; LVEF: Left ventricular ejection fraction; PCI: Percutaneous coronary intervention; SCMR: Society of cardiovascular magnetic resonance; SPECT: Single-photon emission computed tomography.

## Competing interests

The authors declare that they have no competing interest.

## Authors’ contributions

AF conceived the study, participated in its design and coordination, supervised imaging and helped to draft the manuscript. SAA participated in the study design and conduct, and drafted the manuscript. AE participated in the study design and coordination and supervised the performance of the studies. RVS, VD, JC, GB, AAD and RYK participated in the study design and analysis. All authors read and approved the final manuscript.
