# Correction: Impact of cardiovascular magnetic resonance on management and clinical decision-making in heart failure patients

**DOI:** 10.1186/1532-429X-16-20

**Published:** 2014-02-21

**Authors:** Siddique A Abbasi, Andrew Ertel, Ravi V Shah, Vineet Dandekar, Jaehoon Chung, Geetha Bhat, Ankit A Desai, Raymond Y Kwong, Afshin Farzaneh-Far

**Affiliations:** 1 Division of Cardiovascular Medicine, Department of Medicine, Brigham and Women’s Hospital and Harvard Medical School, Boston, MA USA; 2 Section of Cardiology, Department of Medicine, University of Illinois at Chicago, 840 South Wood St. M/C 715, Suite 920 S, Chicago, IL 60612, USA; 3 Division of Cardiology, Department of Medicine, Massachusetts General Hospital and Harvard Medical School, Boston, MA USA; 4 Center for Heart Transplant and Assist Devices, Advocate Christ Medical Center, Oak Lawn, IL USA; 5 Department of Radiology, University of Illinois at Chicago, Chicago, IL USA

## Correction

Following the publication of our article [1] we have noticed that the figure legends for Figure 2 and Figure 3 have been reversed. The figure legends should read as follows:

**Figure 2. Example of a Change in Management.** A 65 year-old man was referred for assessment of ventricular function and viability testing. CMR unexpectedly revealed a large apical thrombus, for which the patient was admitted to hospital for initiation of systemic anticoagulation.

**Figure 3. Example of a New Diagnosis.** A 32 year-old woman with sickle cell anemia was referred for evaluation of iron overload by T2* imaging, which was normal. However, nearly transmural hyperenhancement (white arrows) was seen in the apical inferior wall on late enhancement imaging, indicative of previously unrecognized myocardial infarction.

Please note, these changes do not affect the conclusions made in the original publication.
